# Interference of the Zika Virus E-Protein With the Membrane Attack Complex of the Complement System

**DOI:** 10.3389/fimmu.2020.569549

**Published:** 2020-10-28

**Authors:** Zahra Malekshahi, Britta Schiela, Sarah Bernklau, Zoltan Banki, Reinhard Würzner, Heribert Stoiber

**Affiliations:** ^1^Institute of Virology, Medical University of Innsbruck, Innsbruck, Austria; ^2^Institute of Hygiene & Medical Microbiology, Medical University of Innsbruck, Innsbruck, Austria

**Keywords:** complement, lysis, membrane attack complex, Zika virus, envelope protein, terminal complement pathway

## Abstract

The complement system has developed different strategies to clear infections by several effector mechanisms, such as opsonization, which supports phagocytosis, attracting immune cells by C3 and C5 cleavage products, or direct killing of pathogens by the formation of the membrane attack complex (MAC). As the Zika virus (ZIKV) activates the classical complement pathway and thus has to avoid clearance by the complement system, we analyzed putative viral escape mechanisms, which limit virolysis. We identified binding of the recombinant viral envelope E protein to components of the terminal pathway complement (C5b6, C7, C8, and C9) by ELISA. Western blot analyses revealed that ZIKV E protein interfered with the polymerization of C9, induced on cellular surfaces, either by purified terminal complement proteins or by normal human serum (NHS) as a source of the complement. Further, the hemolytic activity of NHS was significantly reduced in the presence of the recombinant E protein or entire viral particles. This data indicates that ZIKV reduces MAC formation and complement-mediated lysis by binding terminal complement proteins to the viral E protein.

## Introduction

The complement system is an effective arm of innate immunity. It is a family of membrane-anchored and soluble proteins circulating in the blood in their inactive form (1, 2). Upon activation by harmful exogenous or endogenous ligands, one of the three complement pathways is triggered. The classical pathway is induced by immune complexes or by direct binding of C1q to the surface of pathogens, while the lectin pathway is activated by mannose binding lectin (MBL) bound to pathogen-associated molecular patterns, respectively. The third pathway, referred to as the alternative pathway, is initiated by spontaneous hydrolysis of C3 protein. All three pathways result in C3 activation and the formation of C3 convertases, and they merge in the induction of the terminal pathway. This final step generates the membrane attack complex (MAC) consisting of C5b-8 and 12–18 molecules of C9, which makes a pore on the cell surface to kill pathogens or infected cells (1, 2). To avoid destruction by the complement system, viruses have acquired strategies that can be condensed to a few successful mechanisms: 1) inactivation by enzymatic degradation; 2) the recruitment or mimicking of complement regulators; and 3) the modulation or inhibition of complement proteins by direct interactions (3). Different viral families take advantage of at least one of the above-mentioned mechanisms. Among them are retroviruses, orthopox or herpes viruses to name only a few (4–8). In addition, flaviviridae have adapted strategies to escape complement-mediated lysis (9–11).

The flavivirus group includes several human pathogens, such as Dengue (DENV), yellow fever (JFV), West Nile (WNV), Japanese encephalitis (JEV), Zika virus (ZIKV), and the closely related hepatitis C virus (HCV), all of which share close similarities in structure (12, 13). The family belongs to enveloped viruses with a single stranded RNA with positive polarity, which is translated into a single polyprotein. This precursor protein is processed by both host and viral proteases and gives rise to three structural and seven nonstructural (NS) proteins (12, 13). The structural proteins include E protein (envelope protein) PrM, which is the precursor for membrane (M) and plays an important role in virus maturation and capsid (C). The envelope E protein mediates viral entry and modulates infection mainly in its glycosylated form (14). It binds to the different receptors on the surface of human cells and aids in the fusion and subsequent entrance of the virus *via* endocytosis (receptor-mediated endocytosis) (15). NS proteins are responsible for the regulation of RNA transcription, replication, and evasion or attenuation of the host immune response. By NS1, flaviviruses escape from complement-mediated lysis by binding complement regulator proteins such as factor H, C4bp, or vitronectin (9, 10). In line with this, Zika virus (ZIKV) takes advantage of NS1 by binding vitronectin, a regulator protein that interferes with MAC formation by binding to C5, C6, C7, and C9 (16, 17). Furthermore, NS1 may directly reduce C9 polymerization and thus prevent lysis by the terminal pathway complement (18). Although different by mechanism, HCV inhibits C9 polymerization by the acquisition and incorporation of CD59 into the viral envelope (11, 19).

Our data indicates that, similar to NS1, the E protein binds to terminal pathway complement proteins, interferes with the formation of MAC on the surface of the cells and further reduces complement-mediated lysis.

## Materials and Methods

### Proteins

Purified complement proteins C5b6 (MW 285 kDa), C7 (MW 91.4 kDa), C8 (MW 151 kDa), and C9 (MW 71 kDa) were purchased from Complement Technology (Tyler, TX, USA). ZIKV recombinant E and NS1 proteins were provided by Aviva Systems Biology (San Diego, CA); E protein PMA04848-1MG and Biozol (Eching, Germany); E protein MBS596001, and Zika NS1 MBS596002). Anti-flavivirus group antigen antibody [D1-4G2-4-15 (4G2)] and goat anti-mouse antibody conjugated to horseradish peroxidase were purchased from Szabo-Scandic (Vienna, Austria).

Recombinant human vitronectin was obtained from BioLegend (Koblenz, Germany). Bovine serum albumin (BSA) was provided by Carl-Roth (Karlsruhe, Germany).

Anti-C9 (C9 neoantigen, Human, mAb WU13-15) was purchased from Hycult (Uden, NL). Normal human serum (NHS) was acquired from Dunn Labortechnik GmbH (Ansbach, Germany) and stored in aliquots at −80°C. Anti-human HLA-ABC antigen clone W6/32 was obtained from Agilent Technologies Dako (Vienna, Austria). C9 depleted serum was generated as described in detail (20).

### Cells and Viruses

A549 cells for Western blot and virus production and Aedes albopictus C6/36 mosquito cells for virus propagation were kindly provided by Prof. Dr. Karin Stiasny, Medical University of Vienna. Sheep erythrocytes for the hemolysis assay were obtained from Virion (Würzburg, Germany). Two strains of the virus, MRS_OPY_Martinique_PaRi_2015 (GenBank: KU647676) and ZIKV strain MR766 (GenBank: DQ859059) were kindly provided by the European Virus Archive (Marseille, France). The virus was propagated as described elsewhere (21).

### Buffers and Mediums

Cell lysates were analyzed by western blot, RIPA buffer for inducing lysis of the A549 cells was purchased from Cell Signaling Technology (Frankfurt, Germany). Veronal-buffered saline (VBS) was provided by Virion. Dulbecco’s modified Eagle’s medium (DMEM) and phosphate buffered saline (PBS) were purchased from Sigma-Aldrich (Vienna, Austria).

### Binding Assay

Complement proteins including C5b6, C7, C8, and C9 in 1:2 dilutions starting from 5 µg/ml were coated on the microtiter 96 well ELISA plate and incubated overnight at 4°C. After washing with PBS-Tween 0.01%, E protein (10 µg/ml) was added to each well and incubated for 1 h at room temperature (RT) with slow continuous shaking. The ELISA plate was washed three times and blocked with 5% BSA for 1 h. Antibody against envelope protein (4G2) was added at a concentration of 1:500 and incubated for 1 h. Finally, horseradish peroxidase-labeled goat anti-mouse antibody was added (1:10,000). The TMB substrate from Sera Care (Tornesch, Germany) was used. The optical density (OD) was measured at a wavelength of 650 nm. To test if native C9 from NHS was also able to bind to the E protein, serial dilutions of NHS were incubated with a constant amount of E-protein (10 µg/ml coated overnight in the ELISA plate. BSA at the same concentration (10 µg/ml) or dilutions of C9-depleted serum (ΔC9 NHS) were applied as negative controls. Anti-C9 (WU 13-15) at a concentration of 1 µg per well was added. After washing, samples were incubated with secondary antibody (HRP-goat-anti-mouse Ab; 1:10,000) and finally, TMB substrate solution. Optical density (OD) was measured at a wavelength of 650 nm.

### Inhibition of MAC Formation

A C9 polymerization assay was performed to study the formation of a membrane attack complex in the presence or absence of E protein. For this, A549 cells were seeded a day before the experiment at 1 × 10exp5 cells per well in 24 well plates purchased from Szabo-Scandic (Vienna, Austria) in complete DMEM (10% fetal calf serum (FCS; Thermofisher, Vienna, Austria), 2 mM L-glutamine, 100 units/mL penicillin G, 100 µg/ml streptomycin). The next day, cells were washed three times with VBS and C5b6 protein (5 µg in 300 µl) was added to the cells and incubated for 2 h in a humidified incubator supplied with 5% CO_2_ at 37°C. In parallel, the E protein was incubated with 5% NHS at 37°C for 30 min a total volume of 300 µl for each reaction). After three washing steps of the cells with VBS, the mixture of the E protein and NHS was added to the cells and incubated for 60 min at 37°C. Cells were washed again, lysed on ice with RIPA buffer for 30 min (100 µl of lysis buffer), and the lysate was loaded on an 8% acrylamide gel under non-reducing conditions. Lysates were blotted and the membrane was blocked with 5% nonfat dried milk in Tris-buffered saline with 0.1% Tween20 (TBST) for 60 min. The first antibody against C9 protein (WU 13-15) (1:2,000) was added to the blocking solution and incubated overnight at 4°C. The following day, the blot was washed three times with TBST and a horseradish peroxidase-labeled goat anti-mouse antibody was added (1:10,000). After incubation for 2 h at room temperature, the membrane was washed three times and developed using the ImageQuant LAS-4000 (GE Healthcare, Vienna, Austria). In further assays, anti-human HLA-ABC was used in a sublytic amount (1:1,000) as an activator of the classical pathway (instead of purified C5b6 protein). Ab was added to the A549 cells and incubated for 60 min at 37°C. In parallel, different amounts of E protein were incubated with 5% NHS at 37°C for 30 min. After washing the cells with VBS, the mixture of the E protein and NHS was added to the cells and incubated for 50 min at 37°C. Deposition of C9 on the cell surface was analyzed by western blotting as described above.

### Hemolytic Assay

To analyze the activity of the complement system, sheep erythrocytes (1 × 10^8^ cells/ml) resuspended in VBS were sensitized with C5b6 (1 µg) for 60 min at RT using a U-bottom microtiter plate from Greiner Bio-one (Kremsmünster, Austria). In a separate preparation, E protein (10 µg), Vn (20 µg), and mixtures of Vn-and NS1 (containing 20 µg VN and 10 µg NS1) and Vn and-E protein (containing 10 µg E protein and 20 µg Vn) were each incubated with C7-C9 (C7 (1 µg), C8 (0.5 µg), and C9 (1 µg) for 15 min at 37°C. Next, the prepared mixtures were added to the sheep erythrocytes coated with C5b6 and incubated for 30 min at 37°C in a total volume of 100 µl per reaction (9). After centrifugation, the hemolytic activity of the complement system was measured by quantitating the released hemoglobin in the supernatant at 415 nm. To test whether virus particles interfere with complement activation, a two-fold serial dilution of NHS was pre-incubated with ZIKV for 30 min on ice. Sensitized sheep erythrocytes (20 µl, 2 × 10^8^ cells/ml) were added to the samples and the mixture was incubated for 30 min at 37°C. The amount of hemoglobin released from the lysed cells was measured by determining the absorbance of the supernatant at an optical density (OD) of 415 nm. To distinguish between the effects of NS1 or E-Protein on the reduction of hemolysis, viral proteins were incubated separately or as a mixture with NHS (1:160 in VBS) before sensitized sheep erythrocytes were added and the lysis assay was performed as described above.

### Statistical Analyses

Statistical analyses were performed using the GraphPad Prism 7.0 software. All experiments were repeated at least three times always performed in duplicate. The difference between the two groups was assessed by t-test. When comparing more than two groups, ANOVA followed by Bonferroni post-hoc tests was performed. A 95% significance level (p < 0.05) was considered statistically significant (*<0.05, ** <0.01, ***<0.001, and ****<0.001).

## Results

### ZIKV E Protein Binds to Components of the Terminal Pathway of Complement

**Figure 1:**
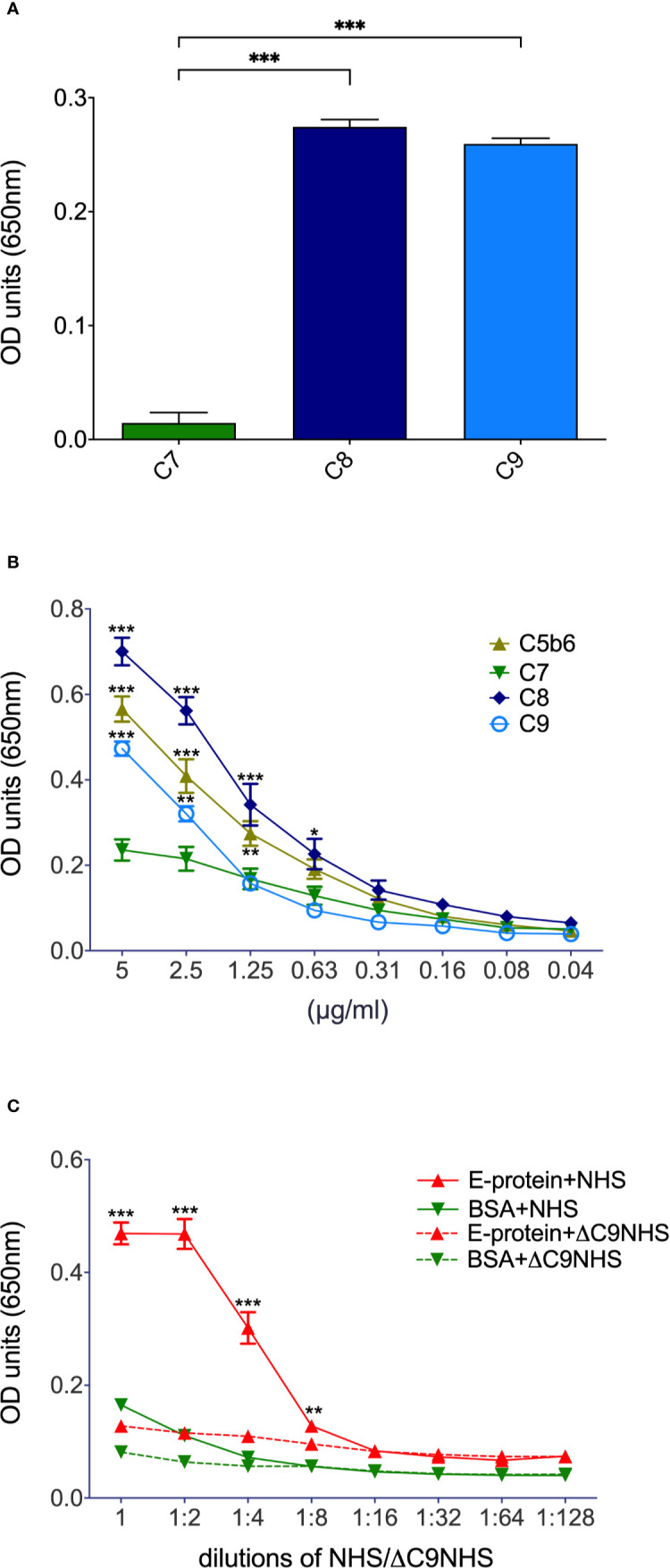
Binding of terminal complement proteins to ZIKV E. Constant amounts (5 µg/ml; **A**) or serial dilutions (**B**, for significance – not always depicted due to limits in space – see text) of purified complement proteins were coated onto ELISA plates and incubated with 10 µg/ml ZIKV E. To visualize binding, an E-specific Ab (4G2) followed by a HRP-goat-anti-mouse Ab and TMB as a substrate were added. To test whether already generated TCC is interacting with ZIKV E too **(C)**, the recombinant viral protein was coated into ELISA plates and incubated with serial dilutions of NHS. BSA and ΔC9 NHS served as controls. Binding to TCC was determined by incubation with neoepitope-specific anti-C9 (WU 13-15) followed by HRP-goat-anti-mouse Ab. Again, TMB was used as a substrate. Optical density (OD) was measured at a wavelength of 650 nm. Experiments were repeated three times and were performed in duplicates. For statistical analysis GraphPad Prism software was used (**A**, 1-way ANOVA; **B, C**, 2-way ANOVA, respectively). *< 0.05, ** < 0.01 and *** < 0.001.

As ZIKV activates the classical pathway of complement, we were interested in whether the virus adapted means to reduce virolysis. In a first attempt, we assessed whether purified C7, C8, or C9 bind ZIKV E in ELISAs. In contrast to C7, both C8 and C9 interacted with the viral recombinant E protein (**Figure 1A**). Both C8 and C9, but also C5b6 dose-dependently bound to ZIKV E protein (**Figure 1B**). Significance was reached for C8 down to 0.63 µg/ml of ZIKV E (**Figure 1B**), and for C9 and C5b6 down to 1.25 µg/ml. Finally, the binding of ZIKV E protein to the already generated terminal complement cascade (TCC) was assessed in NHS. For this, a constant amount of ZIKV E or BSA was coated onto the ELISA plates and incubated with different dilutions of NHS. As a further control, ΔC9 NHS was included. Significant interaction of TCC with the viral protein was observed up to an NHS dilution of 1:8 compared to the ΔC9 NHS (**Figure 1C**). With regard to BSA, only background binding to TCC was detected, even at the highest concentration of NHS (**Figure 1C**).

### ZIKV E Protein Reduces C9 Polymerization on Cellular Surfaces

**Figure 2:**
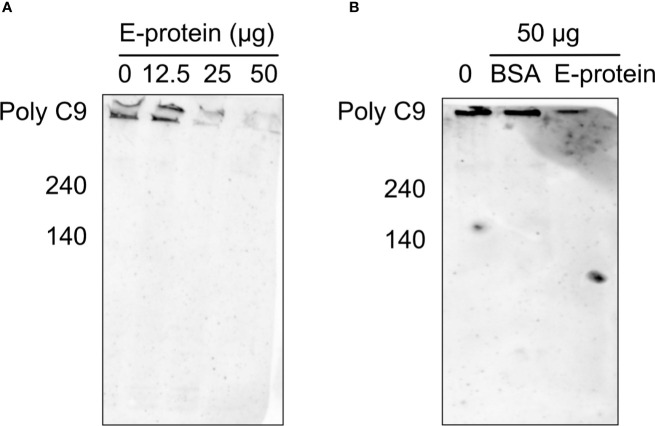
Inhibition of C9 polymerization after induction *via* C5b6 in the presence of ZIKV E. To trigger generation of the terminal pathway of complement, A549 cells were incubated with purified C5b6 (5 µg) for 2 h at 37°C. After washing, 5% NHS was added as a source of C7 to C9 in the presence of different amounts of ZIKV E. After incubation at 37°C for 50 min and additional washing steps, cells were lysed and loaded on a SDS gels under non-reducing conditions. Lysates were blotted and visualized as described in the Material & Method section. A representative Western blot out of three independent experiments is shown **(A)**. As a control, the effect of BSA on the C9 polymerization was used **(B)**.

**Figure 3:**
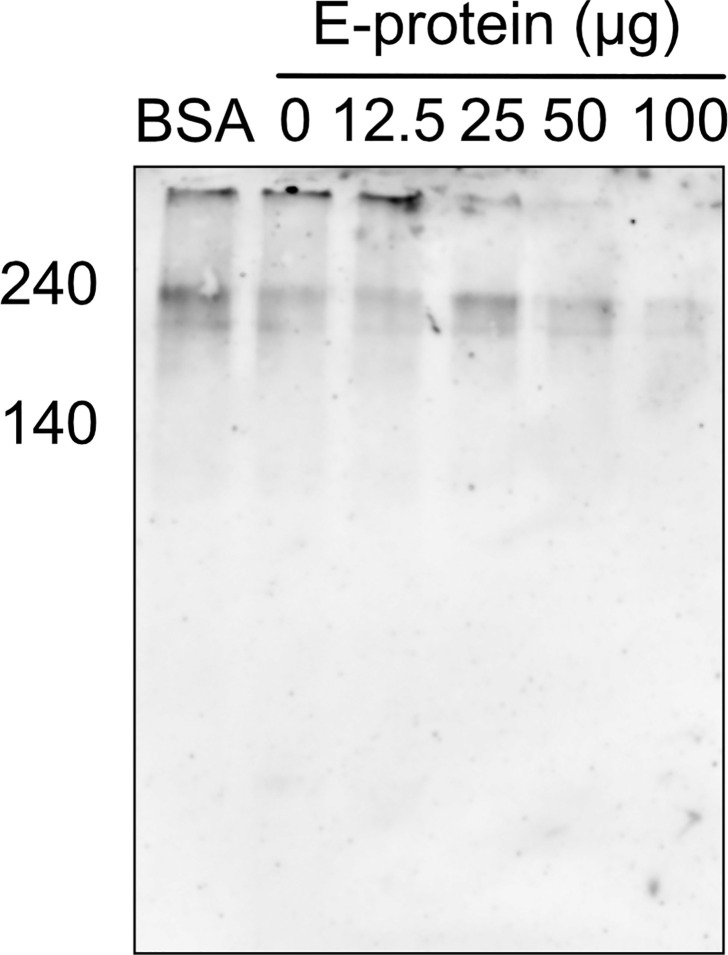
Inhibition of C9 polymerization after induction of the classical pathway in the presence of ZIKV E. For the induction of complement activation, A549 cells were incubated with sublytic amounts of anti-MHC-1 (1:1,000). After washing, cells were incubated with 5% NHS, which was pre-incubated with different amounts of ZIKV E. After washing, cells were blotted and oligomerization of C9 was analyzed as described in **Figure 2**. A representative Western blot out of three independent experiments is shown.

To test whether the binding of ZIKV E to components of the TCC interferes with the polymerization of C9, A549 cells were incubated with purified C5b6. As a source of C7 to C9, 5% NHS was used in the absence (**Figure 2**; 0 = no E) or the presence of different amounts of ZIKV E (**Figure 2**, 12.5 to 50 µg). The polymerization of high molecular weight C9 at the cellular surface was confirmed by western blot of the cell lysates employing the C9 neo-epitope-specific anti-C9 antibody (WU 13-15). Polymeric C9 was markedly reduced in a ZIKV E-concentration-dependent manner (**Figure 2A**), while BSA had no effect (**Figure 2B**) To further analyze the effect of ZIKV E on C9 polymerization, A549 cells were first incubated with sublytic amounts of an anti-MHC-I antibody as a trigger for the classical complement pathway. After removing the antibody by washing, NHS was added to the cells, which were pre-incubated with different amounts of ZIKV E. Cell lysates were analyzed by western blotting. C9 polymerization was reduced in a dose-dependent manner (**Figure 3**). However, in contrast to the induction of TCC by incubation of the cells with purified C5b6, which gave rise to high-molecular-weight C9 polymers, the activation of the classical pathway of complement induced C9 oligomers of about 210 kDa in size (**Figure 3**). Again, the band observed for high molecular C9 polymers were reduced when compared to BSA.

### ZIKV E Protein Inhibits the Formation of a Membrane Attack Complex

**Figure 4:**
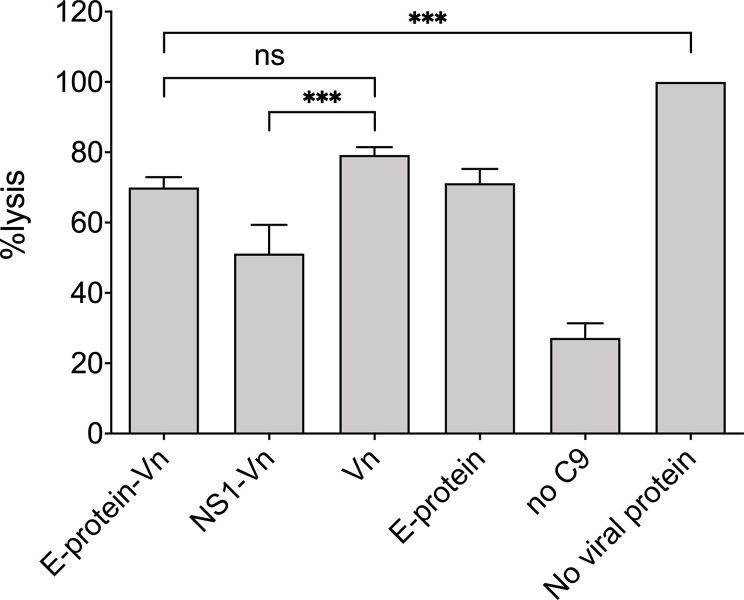
Inhibition of MAC formation by ZIKV E: Viral proteins were pre-incubated with C7 to C9 and added to sheep erythrocytes pre-coated with C5b6. As a control vitronectin (VN) was used, a known inhibitor of the MAC. In the positive control (no viral protein) formation of the MAC was undisturbed and set at 100%. Hemolysis was determined by measuring the release of hemoglobin in the supernatant at an OD of 415 nm. Omission of C9 served as background control. Experiments were repeated three times performed in duplicates and analyzed by 1-way ANOVA. n.s., not significant. *** < 0.001.

The interference of ZIKV E with the proteins of the terminal complement pathway might affect complement-mediated lysis, similar to that described for other pathogens (18, 22–24). Therefore, as sensitive functional readout, hemolytic assays with sheep erythrocytes were performed using purified TCC components. For this, ZIKV E was pre-incubated with C7, C8, and C9 and added to C5b6 pre-coated erythrocytes. When compared to lysis of the cells in the absence of viral proteins, which was set at 100% lysis, ZIKV E significantly reduced hemolysis (**Figure 4**) similar to that observed for vitronectin, a known inhibitor of the TCC (16). We confirmed that also NS1 interferes with complement-mediated hemolysis (not shown) and reproduced the data of Conde and coworkers, who showed a synergistic effect of NS1 with vitronectin (18). This synergy was not observed by combining ZIKV E with vitronectin (**Figure 4**).

**Figure 5:**
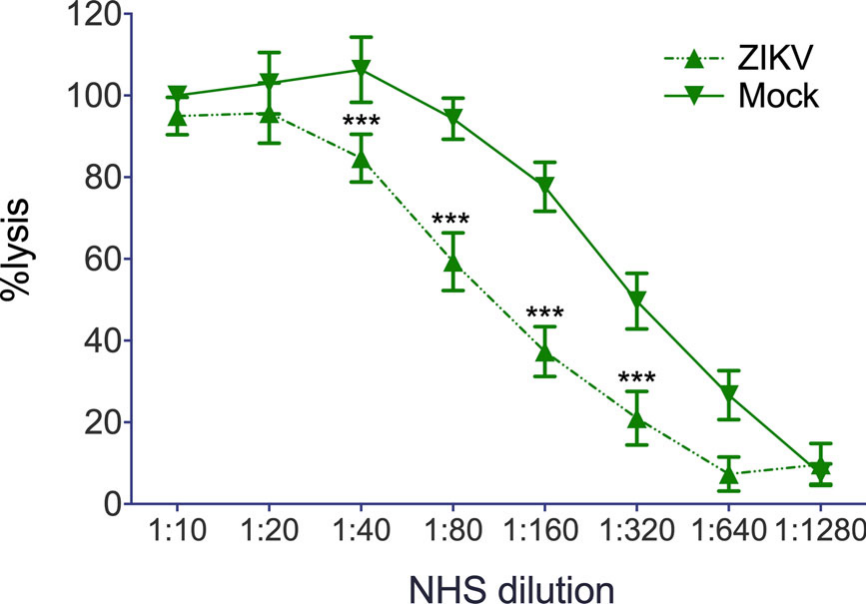
Reduction of complement mediated lysis by ZIKV: Sensitized sheep erythrocytes were incubated for 30 min at 37°C with serial dilutions of NHS. As expected, lysis of the cell decreased with decreasing dilutions of NHS and was not affected by DMEM, the buffer used as mock control. Hemolysis was determined by measuring the release of hemoglobin in the supernatant at 415 nm. Experiments were performed three times in triplicates. Significance was calculated by 2-way ANOVA. *** < 0.001.

**Figure 6:**
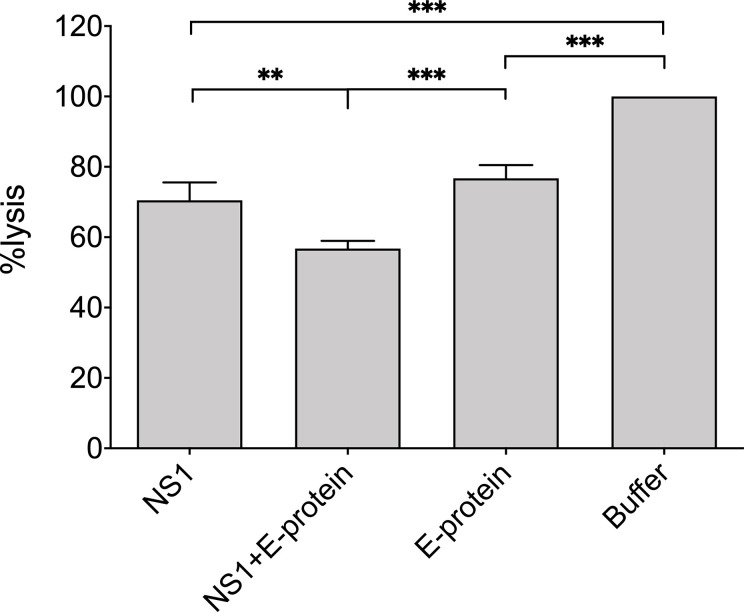
Reduction of complement mediated lysis by recombinant viral proteins: NHS (1:160 diluted in VBS) was incubated on ice with purified recombinant viral proteins E (20 µg) or NS1 (5 µg) or a mixture of both. Sensitized sheep erythrocytes were added and incubated for 30 min at 37°C. The VBS-buffer control was set at 100%. Hemolysis was determined by measuring the release of hemoglobin in the supernatant at 415 nm. Experiments were performed three times in duplicates and analyzed by 1-way ANOVA. ** < 0.01 and *** < 0.001.

Finally, we were interested in whether not only recombinant viral proteins, but also ZIKV itself interferes with hemolysis of sensitized erythrocytes. Erythrocytes were lysed by NHS in a dose-dependent manner and were not affected by the mock control, which employs the DMEM buffer used to cultivate the cells for virus propagation (**Figure 5**). In contrast, hemolysis was significantly diminished when ZIKV (2.5 × 10 exp5 PFU) was present in the system, starting from a 1:40 dilution of NHS (**Figure 5**), indicating that not only recombinant viral proteins, but also viral particles interfere with MAC formation. To check whether NS1 and the E protein show additive effects, the viral protein was incubated separately and as a mixture with NHS before the erythrocytes were added. As expected, both viral proteins reduced hemolysis when compared to the buffer control (VBS). The mixture of NS1 and E further decreased cell lysis, indicating that both viral proteins contribute to the inhibition of complement-mediated lysis (**Figure 6**).

## Discussion

Interference with complement-mediated lysis is a common complement evasion mechanism for many viruses (9, 10). Thus, it is no surprise that members of the flavivirus family have developed strategies to escape from virolysis by interacting with proteins of the complement cascades and their regulators with NS1. WNV, for example, binds factor-H (25), a regulator of complement in the fluid phase, which interferes with the convertases and acts as a co-factor for C3b inactivation (1, 2). C4b binding protein (C4bp), a regulator of the classical and lectin pathway, is not only recruited by DENV (26), but also by WNV or YVF (27). Furthermore, the viruses’ complex C4 together with C1s/proC1s in the fluid phase to decrease C4b deposition on the viral surface. Consequently, the classical pathway convertase is reduced and less MAC is induced (22). Clusterin (28) and vitronectin (18), two inhibitors of the TCC can interfere with MAC-formation by binding to NS1. In addition, flaviviral NS1, including ZIKV, can directly decrease C9 polymerization on cell surfaces and thus evade complement-induced damage (18). As mentioned above, these different strategies are attributed to the NS1 protein. Here, we report that besides NS1 also the E protein of ZIKV can directly interact with proteins of the TCC. In contrast to NS1, which also binds to C7, besides C5, C6, and C9, but not C8 (18), the viral E-protein was capable of interacting with C8, and only a poor interaction with C7 was observed. Consequently, the polymerization of C9 was reduced by the E protein in a dose-dependent manner when purified proteins were used. Of note, at basis of the molecular weight, more C9 was necessary to bind the same amount of ZIKV E. Thus, it would be interesting to check the effect of ZIKV E on the association of C8 or C5b6 during MAC formation. However this is beyond the scope of this paper. Activation of the classical pathway by antibodies bound to the cell surface resulted in a decrease of C9 oligomers in the presence of ZIKV E. Beside the bands for high molecular polymers, additional bands were identified compared to that of the experiments, in which the purified components were used. This corresponded to the size of trimerized C9 oligomers, which could be formed due to sublytic amounts of antibody used for complement activation.

According to our data, ZIKV E interfered with complement-mediated hemolysis comparable to vitronectin. Although interacting with vitronectin (data not shown), lysis induced by E protein was not further enhanced when both proteins were co-applied. In contrast, hemolysis was enhanced by vitronectin when the protein was added together with NS1, which confirms the recently published data of Conde et al. (18). As we were interested in whether lysis was impaired not only by purified complement proteins, but also in NHS as a source of complement, hemolysis assays were performed in the presence of ZIKV particles. Indeed, about four times more NHS was needed for complement-induced lysis of the cells when ZIKV was present. However, this experiment does not allow us to distinguish whether this effect is attributed to the E protein, NS1, or both. Therefore, purified recombinant viral proteins were used. Hemolysis assays showed that NS-1 and E proteins have additive effects, and thus, both proteins may contribute to the reduction in complement activity. Of note, more ZIKV E protein than NS1 was necessary to show comparable effects in the hemolysis assay, which might be due to a higher affinity of NS1 to proteins of the terminal pathway.

In summary, not only NS1, but also ZIKV E protein can reduce the formation of the MAC. As ZIKV activates the classical pathway by direct binding of C1q to the E protein and infection by this virus upregulates the expression of complement proteins (29), the virus has adopted several strategies to interfere with complement attack assembly. This corroborates the view that multiple evasion strategies are used by microorganisms, and in particular viruses, to limit damage by the complement system.

## Data Availability Statement

The original contributions presented in the study are included in the article/supplementary material. Further inquiries can be directed to the corresponding authors.

## Author Contributions

ZM, BS, and SB: experimental work. ZB, RW, and HS: study design, data interpretation, drafting the article, critical revision of the article, and final approval. All authors contributed to the article and approved the submitted version.

## Funding

This study was supported by the FWF, Vienna Austria (HOROS doctoral Program, W-1253 DK HOROS).

## Conflict of Interest

The authors declare that the research was conducted in the absence of any commercial or financial relationships that could be construed as a potential conflict of interest.
